# Retinal Neurovascular Changes in Patients With Ischemic Stroke Investigated by Optical Coherence Tomography Angiography

**DOI:** 10.3389/fnagi.2022.834560

**Published:** 2022-07-04

**Authors:** Yingying Liang, Baoyi Liu, Yu Xiao, Xiaomin Zeng, Guanrong Wu, Zijing Du, Ying Fang, Yijun Hu, Xiaohong Yang, Honghua Yu

**Affiliations:** ^1^Guangdong Eye Institute, Department of Ophthalmology, Guangdong Provincial People’s Hospital, Guangdong Academy of Medical Sciences, The Second School of Clinical Medicine, Southern Medical University, Guangzhou, China; ^2^State Key Laboratory of Ophthalmology, Zhongshan Ophthalmic Center, Sun Yat-sen University, Guangzhou, China; ^3^School of Medicine, South China University of Technology, Guangzhou, China

**Keywords:** neurovascular, retinal vessel density, ganglion cell complex, retinal nerve fibre layer, ischemic stroke, optical coherence tomography angiography (OCTA)

## Abstract

**Background:**

To investigate retinal neurovascular structural changes in patients with ischemic stroke (IS) using optical coherence tomography angiography (OCTA).

**Materials and Methods:**

The cross-sectional study was conducted in Guangdong Provincial People’s Hospital, China, consisting of 159 eyes from IS patients and 109 eyes from age-matched control subjects. Retinal microvascular parameters including the vessel density (VD) of the superficial capillary plexus (SCP), deep capillary plexus (DCP) and radial peripapillary capillary (RPC), and neural parameters such as ganglion cell complex thickness (GCCt) and retinal nerve fibre layer thickness (RNFLt) were measured by OCTA.

**Results:**

The VD of SCP and DCP in the macular area were significantly reduced in IS patients compared to the control group (all *p* < 0.001). The VD of RPC at the optic disc was also significantly reduced in IS patients (all *p* < 0.05). IS patients showed reduced GCCt and RNFLt and increased GCC focal loss volume and global loss volume compared with the controls (all *p* < 0.05). Among patients with IS, the parafovea SCP VD was positively correlated with GCCt (*r* = 0.346–0.408, all *p* < 0.001) but not with DCP VD (all *p* > 0.1). In the optic disc region, the whole image RPC VD was positively correlated with mean RNFLt (*r* = 0.467–0.548, all *p* < 0.001).

**Conclusion:**

Reduction of retinal VD, GCCt and RNFLt was observed in patients with IS. The parafovea SCP VD and RPC VD were positively correlated with GCCt and RNFLt, respectively.

## Introduction

Stroke is the second cause of mortality globally (Naghavi’s et al., 2017) and the leading cause of mortality and adult disabilities in China (GBD 2019 Stroke Collaborators, 2021). China Stroke Statistics 2019 estimated that the age-standardized prevalence and incidence of stroke were 1114.8 per 100000 population and 246.8 per 100000 person-years, respectively (Wang et al., 2020). In the clinic, stroke is presented in two major forms: ischemic stroke (IS) and hemorrhagic stroke (HS), of which IS accounts for 80% (Dichgans, 2007). IS has the characteristics of high morbidity, disability, mortality, and high recurrence rate, posing great burden on individuals and society (Roth et al., 2020). The burden of IS is expected to be further increased as a consequence of population aging and an ongoing high prevalence of risk factors (e.g., hypertension).

In previous studies, sublingual microcirculation (Khalilzada et al., 2011; Martens et al., 2013) and the indicators of neurological function in the serum including neuron-specific enolase, S100B protein and glial fibrillary acidic protein have been used to evaluate the microcirculation and neurological function in patients with cerebrovascular disorders (Liu and Geng, 2018; Lasek-Bal et al., 2019). In recent years, many studies have also focused on the change of retinal neurovascular structures in cerebrovascular diseases. As is well known, the retina is an extension of the central nervous system (CNS), possessing many similarities with the brain in terms of developmental origin, anatomical features, and physiological properties (London et al., 2013; Ma Cc Ormick et al., 2015). Both the retina and CNS have similar microvascular architecture, autoregulation of blood flow, vascular barrier function, and the important homeostatic role of neurovascular coupling responses (Kur et al., 2012; London et al., 2013). Thus, the retina may provide a direct, non-invasive, and easily accessible window for observing the neurovascular structures of the CNS.

In the past, most research was based on color fundus photography to observe morphologic changes of retinal vessels in IS patients, such as narrowing of arterial diameter, thickening of venous diameter and arteriovenous impressions (Yatsuya et al., 2010; Kawasaki et al., 2012; Cheung et al., 2017). Optical coherence tomography was also applied to investigate the focal loss of nerve fiber layer in patients with IS (Wang et al., 2014). With advances in retinal imaging technology, the high contrast and resolution in optical coherence tomography angiography (OCTA) images make it possible to accurately quantify the retinal microvasculature and neural structural changes in vivo simultaneously. Recently, OCTA has been used to observe the retinal microvascular and neural alterations in systemic diseases, including diabetes, hypertension, and Alzheimer’s disease (Sun et al., 2019; Chua et al., 2020; Peng et al., 2020). There are also several studies in which OCTA was used to observe the changes of retinal microcirculation in stroke patients (McGrory et al., 2019; Zhang X. et al., 2020; Liu et al., 2021). However, the relationship between retinal microvascular and neural changes is unknown. Therefore, in the present study, we aimed to investigate retinal microvascular and neural alterations and their associations in patients with IS using OCTA.

## Materials and Methods

### Design and Study Participants

In this cross-sectional retrospective study, participants with IS were included from the Department of Neurology of Guangdong Provincial People’s Hospital (GDPH) from May 2019 to December 2019. A diagnosis of ischemic stroke was confirmed for all patients by history, clinical symptoms, signs, computed tomography (CT) or magnetic resonance imaging (MRI). We divided IS patients into anterior circulation stroke (ACS) and posterior circulation stroke (PCS) according to the lesion location shown in digital subtraction angiography (DSA), CT or MRI. We excluded patients with eye diseases affecting OCTA parameters (such as uveitis, glaucoma, age-related macular degeneration, diabetic retinopathy), history of ophthalmic surgery, a refractive error (higher than +3.0 diopter or lower than –3.0 diopter), or poor OCTA quality on both eyes.

Volunteers without either a history of IS or ocular diseases were recruited as controls. Both groups were matched for age. The study was approved by the ethics commission of GDPH and followed the Declaration of Helsinki. All participants gave written informed consent.

### Ophthalmic Examinations

The participants underwent the best-corrected visual acuity (BCVA) measurements, non-contact intraocular pressure (IOP), slit-lamp examinations, color fundus photography and OCTA examinations (RTVue-XR Avanti; Optovue, Fremont, CA, United States). The OCTA examinations, using high definition modes of the optic disc (4.5 × 4.5mm^2^) and macula (6 × 6mm^2^), were performed with patients’ pupils dilated.

Vessel density (VD) was defined as the percentage of signal positive pixels per total pixels in an area of interest. The software sets the superficial capillary plexuses (SCP) from 3μm below the internal limiting membrane (ILM) to 15μm below the inner plexiform layer (IPL). The deep capillary plexus (DCP) was set from 15 to 70μm below the ILM (Zeng et al., 2021). In addition, the 1- to 3-mm annulus foveal region will be referred to as the parafovea, and the 3- to 6-mm annulus as the perifovea. The foveal avascular zone (FAZ) area was automatically measured by the AngioVue software. In the disc area, the boundary of radial peripapillary capillary (RPC) ranges from ILM to the nerve fiber layer. The peripapillary region is defined as a 1–2 mm round annulus around the optic disc, while the capillary density was measured with automatic removal of larger vessels (diameter ≧33 μm) (Campbell et al., 2017).

The ganglion cell complex thickness (GCCt) was the inner retinal thickness values from ILM to IPL. GCC focal loss volume (FLV) and GCC global loss volume (GLV) were automatically calculated using the AngioVue software. The retinal nerve fibre layer thickness (RNFLt) was measured by a circle scan 3.45 mm in diameter centered on the nerve, which was divided automatically into superior-hemifield and inferior-hemifield (Cennamo et al., 2020).

Both eyes of the participants were examined using OCTA, but only the data of the right eye were included. If the scan of the right eye was uninterpretable, data of the left eye were used for analysis. Only images with quality index ≥ 6 were retained, considering that OCTA measurement of the VD and neural structure can be affected by media opacity (Cheung et al., 2012; Zhang J. et al., 2020).

### Demographic, Clinical and Laboratory Data Collection

Demographic, clinical and laboratory data were extracted and collected from the hospital registration system by trained research assistants and ophthalmologists. The demographic and clinical data included age, gender, previous history (hypertension, diabetes mellitus, smoking), systolic blood pressure (SBP), diastolic blood pressure (DBP), CT and MRI. Laboratory test data included fasting blood glucose (FBG), glycated hemoglobulin (HbA1c), total cholesterol (CHOL), triglyceride (TRIG), high-density lipoprotein (HDL), low-density lipoprotein (LDL).

### Statistical Analysis

Statistical Product and Service Solutions (SPSS) version 26.0 (SPSS. Lnc., Chicago, IL, United States) was used to perform statistical analyses. Continuous variables are displayed as means and standard deviation (SD) or medians and interquartile range (IQR). Categorical variables are presented as numbers and percentages. Data normality were assessed by the Shapiro-Wilk test. Basic characteristics between IS patients and control subjects were analyzed by two-tailed independent Student’s *t*-test or Mann-Whitney test. Analysis of covariance (ANCOVA) was applied to compare the OCTA parameters while adjusting the significantly different baseline characteristics between the two groups. Partial correlation analyses between retinal VD and thickness parameters were performed after adjusting the covariates. OCTA parameters were analyzed by using binary logistic regression analysis to assess the risk factors for IS. Subgroup analyses of IS group were performed. The *p* values were expressed as outcomes and a *p* < 0.05 was considered as statistically significant.

## Results

### Study Population

Overall, a total of 268 participants including 159 IS patients and 109 age-matched control subjects were recruited. The IS group had a mean age of 60.5 (11.4) years and the control group had a mean age of 58.5 (10.0) years. The demographic and clinical characteristics of the two groups are shown in Table 1. There were significant differences between the two group in sex (*p* < 0.001), SBP (*p* < 0.001), DBP (*p* < 0.001), history of smoking (*p* < 0.001), HbA1c (*p* = 0.038), CHOL (*p* = 0.001), HDL (*p* < 0.001), and BCVA (*p* < 0.001).

**TABLE 1:** Demographic and clinical characteristics of the two groups.

	Ischemic stroke	Controls	*P-value*
Number of eyes (patients)	159	109	N/A
Age(y), mean(SD)	60.5(11.4)	58.5(10.0)	0.138
Sex, female, n(%)	39(24.5)	65(59.6)	<0.001*
SBP, mmHg, mean(SD)	145.1(21.7)	130.3(15.0)	<0.001*
DBP, mmHg, mean(IQR)	83.0(14.0)	75.0(13.0)	<0.001*
History of HTN, n(%)	73(45.9)	46(42.2)	0.548
History of DM, n(%)	77(48.4)	41(37.6)	0.080
History of smoking, n(%)	51(32.1)	11(10.1)	<0.001*
FBG(mmol/L), mean(IQR)	5.8(1.1)	5.7(0.9)	0.297
HbA1c(mmol/L), mean(IQR)	6.1(1.2)	5.7(0.6)	<0.001*
CHOL(mmol/L), mean(IQR)	4.3(1.5)	4.7(1.3)	<0.001*
TRIG(mmol/L), mean(IQR)	1.6(0.9)	1.5(0.8)	0.442
HDL(mmol/L), mean(IQR)	1.0(0.3)	1.2(0.3)	<0.001*
LDL(mmol/L), mean(IQR)	2.8(1.0)	2.5(0.9)	0.373
IOP(mmHg), mean(IQR)	14.3(3.9)	14.8(3.6)	0.145
BCVA (logMAR), mean(IQR)	0.1(0.2)	0.0(0.1)	<0.001*
Quality Index, mean(IQR)	8(2)	8(2)	0.081

*SBP, systolic blood pressure; DBP, diastolic blood pressure; HTN, hypertension; DM, diabetes mellitus; FBG, fasting blood glucose; HbA1c, hemoglobin A1C; CHOL, cholesterol; TRIG, triglycerides; HDL, high-density lipoprotein; LDL, low-density lipoprotein; BCVA, the best-corrected visual acuity; IOP, intraocular pressure.*

*p < 0.05 is considered statistically significant.

The time gap between the stroke attack and the OCTA examination was 1-372 days in the IS group with the median of 16 days. No significant differences in OCTA parameters between IS patients with shorter time interval and those with longer time interval were observed. OCTA parameters in the ischemic stroke group were not correlated to the time interval between ischemic stroke attacks and OCTA examination (Supplementary Tables 1, 2).

### Comparison of Retinal Microvascular Parameters

Significant differences were found in both SCP and DCP VD between the two groups. In the SCP, the whole image, parafoveal and perifoveal VD were 47.8%, 49.9%, and 48.6%, respectively, in the IS patients, and 49.7%, 52.8%, and 50.6%, respectively, in the control group (Table 2). In the DCP, the whole image, parafoveal and perifoveal VD were 48.3%, 52.8%, and 49.4%, respectively, in the IS patients, and 51.4%, 55.6%, and 52.8%, respectively, in the control group (Table 2). The SCP and DCP VD were significantly lower in patients with IS compared to controls (all *p* < 0.05).

**TABLE 2:** Comparison of optical coherence tomography angiography (OCTA) parameters in the two groups.

	Ischemic stroke	Controls	*P-value* ^#^
**Retinal microvascular parameters**			
*SCP VD (%), mean(SD)*			
Whole image	47.8(4.0)	49.7(3.6)	<0.001*
Parafovea	49.9(5.0)	52.6(4.1)	<0.001*
Perifovea	48.6(4.2)	50.6(3.7)	<0.001*
*DCP VD (%), mean(SD)*			
Whole image	48.3(5.6)	51.4(5.7)	<0.001*
Parafovea	52.8(4.4)	55.6(3.8)	<0.001*
Perifovea	49.4(6.2)	52.8(6.1)	<0.001*
*RPC VD (%), mean(SD)*			
Whole image	49.1(2.5)	49.8(2.4)	0.006*
Peripapillary	51.2(2.6)	52.0(2.9)	0.043*
S-Hemi	51.5(2.9)	52.0(3.0)	0.021*
I-Hemi	51.1(3.1)	52.2(2.7)	0.010*
**Retinal neural parameters**			
*GCC, mean(SD)*			
Whole image, μm	98.7(8.5)	101.4(6.7)	0.024*
Parafovea, μm	105.3(9.7)	107.7(7.1)	0.024*
Perifovea, μm	98.8(8.6)	101.6(7.2)	0.032*
FLV,%	1.2(1.3)	0.7(0.6)	0.001*
GLV,%	3.0(2.9)	1.7(1.8)	0.002*
*RNFL(μm), mean(SD)*			
Mean	113.3(12.6)	116.1(12.1)	0.017*
S-Hemi	114.1(12.0)	116.4(13.0)	0.023*
I-Hemi	112.1(13.9)	116.0(12.1)	0.005*

*VD, vessel density; SCP, superficial capillary plexus; DCP, deep capillary plexus; RPC, radial peripapillary capillaries; GCC, ganglion cell complex; RNFL, retinal nerve fibre layer; FLV, focal loss volume; GLV, global loss volume; S-Hemi, superior-hemifield; I-Hemi, inferior-hemifield.*

*^#^Adjusted for sex, SBP, DBP, history of smoking, HbA1c, CHOL, HDL, BCVA of enrolled eyes.*

*p < 0.05 is considered statistically significant.

The FAZ area of IS subjects was 0.32 ± 0.10 and the FAZ area of the controls was 0.35 ± 0.10. No significant differences were found in the FAZ area between the two groups (*p* = 0.141).

A significant reduction in RPC VD (whole image, peripapillary, superior-hemifield and inferior-hemifield) was found in patients with IS compared to controls (Table 2).

**FIGURE 1:**
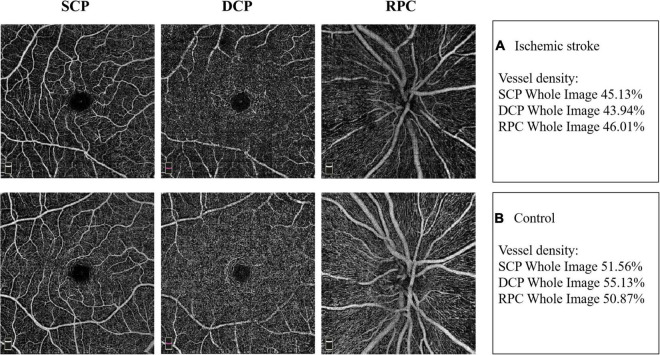
Example images of retinal vascular density in the two groups. **(A)** The retinal vessel density in superficial capillary plexus (SCP), deep capillary plexus (DCP) and radial peripapillary capillaries (RPC) in IS patient. **(B)** The retinal vessel density in SCP, DCP and RPC in non-stroke subject.

Typical images of retinal VD in SCP, DCP and RPC of the two groups were presented in Figure 1.

### Comparison of Retinal Neural Parameters

There were significant differences in whole image, peripapillary, superior-hemifield and inferior-hemifield GCCt between the two groups (all *p* < 0.05). In addition, FLV and GLV of the GCC were found to be increased in IS patients. A significant decrease in the RNFLt of mean, superior-hemifield and inferior-hemifield sectors was found in the IS group compared to the control group (Table 2).

**FIGURE 2:**
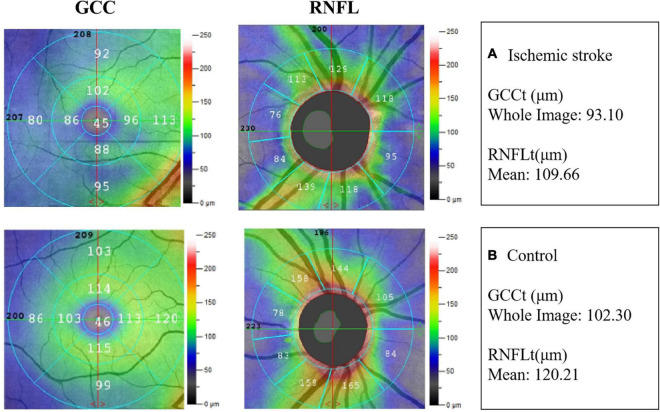
Example images of retinal neural thickness in the two groups. **(A)** The ganglion cell complex thickness (GCCt) and retinal nerve fibre layer thickness (RNFLt) in IS patient. **(B)** The GCCt and RNFLt in non-stroke subject.

Typical images of GCCt and RNFLt of the two groups were presented in Figure 2.

### Correlation Analyses Between Retinal Microvascular Parameters and Neural Parameters

In the partial correlation analyses in the IS group, after adjusting for sex, SBP, DBP, history of smoking, HbA1c, CHOL, HDL, and BCVA, the parafoveal SCP VD was positively correlated with whole image GCCt (*r* = 0.408, *p* < 0.001), parafoveal GCCt (*r* = 0.346, *p* < 0.001) and perifoveal GCCt (*r* = 0.389, *p* < 0.001). Meanwhile, the parafoveal SCP VD showed a weak negative correlation with GLV (*r* = –0.181, *p* = 0026). There were no significant correlations between the parafoveal DCP VD and GCCt. In the optic disc region, the whole image RPC VD was positively correlated with mean RNFLt (*r* = 0.548, *p* < 0.001), superior-hemifield RNFLt (*r* = 0.467, *p* < 0.001), and inferior-hemifield RNFLt (*r* = 0.563, *p* < 0.001). Details about the correlations are shown in Table 3.

**TABLE 3:** The correlation between microvascular parameters and neural parameters.

	Correlated Parameters	*r* ^#^	*P-value* ^##^
SCP-Parafovea	Whole Image GCC	0.408	<0.001*
	Parafovea GCC	0.346	<0.001*
	Perifovea GCC	0.389	<0.001*
	GCC-FLV	–0.112	0.173
	GCC-GLV	–0.181	0.026*
DCP-Parafovea	Whole Image GCC	0.067	0.415
	Parafovea GCC	0.061	0.453
	Perifovea GCC	0.074	0.363
	GCC-FLV	0.029	0.720
	GCC-GLV	0.014	0.866
RPC-Whole image	Mean RNFL	0.548	<0.001*
	S-Hemi RNFL	0.467	<0.001*
	I-Hemi RNFL	0.563	<0.001*

*SCP, superficial capillary plexus; DCP, deep capillary plexus; RPC, radial peripapillary capillaries; GCC, ganglion cell complex; RNFL, retinal nerve fibre layer; FLV, focal loss volume; GLV, global loss volume; S-Hemi, superior-hemifield; I-Hemi, inferior-hemifield.*

*^#^r indicates the partial correlation coefficient between microvascular parameters and neural parameters.*

*^##^Adjusted for sex, SBP, DBP, history of smoking, HbA1c, CHOL, HDL, BCVA of enrolled eyes.*

*p < 0.05 is considered statistically significant.

For control group, the parafoveal SCP VD was positively correlated with whole image GCCt (*r* = 0.228, *p* = 0.022), parafoveal GCCt (*r* = 0.256, *p* = 0.010) and perifoveal GCCt (*r* = 0.223, *p* = 0.025) after adjusting for sex, SBP, DBP, history of smoking, HbA1c, CHOL, HDL, and BCVA. There were no correlations between the parafoveal DCP VD and GCCt, nor between the whole image RPC VD and RNFLt (Supplementary Table 3).

### Logistic Regression Analysis

The binary logistic regression analysis showed that participants with higher VD of SCP, DCP and RCP (OR: 0.848–0.978, all *p* < 0.05 except for superior-hemifield RPC VD), higher GCCt (OR: 0.956–0.969, *p* < 0.05) and higher inferior-hemifield RNFLt (OR = 0.978, *p* = 0.020) were found to have lower odds of being classified as stroke, while higher FLV (OR = 1.795, *p* < 0.001) and GLV (OR = 1.249, *p* < 0.001) were found to have higher odds of the participants being classified as stroke (Supplementary Table 4).

### Subgroup Analyses

Significant differences were found in both whole image and perifoveal VD of SCP and DCP between ACS and PCS. Meanwhile, there were significant differences in whole image and peripapillary GCCt between the two types of stroke. No significant differences were found in any sections of RCP VD or RNFLt (Supplementary Table 5).

The ANCOVA results conducted to compare the OCTA parameters between subjects with diabetes and without diabetes in the IS group showed no significant differences in OCTA parameters between subjects with diabetes and without diabetes (all *p* < 0.05).

There were no significant differences in OCTA parameters between female and male subjects in the IS or control group (Supplementary Tables 6, 7).

## Discussion

Retina is considered an extension of the brain and thus serves as a gateway to the brain. In the present study, to investigate the neurovascular impairment in patients with IS, OCTA was used to observe retinal microvascular and neural alterations. Results of our study showed that VD in the macular and optic disc areas was significantly reduced in patients with IS, and a decrease in RNFLt and GCCt was also detected. Meanwhile, there were significant correlations between retinal microvascular and neural alterations in IS patients.

A significant reduction was found in the VD of the macular and optic disc areas, which was similar with the results of a previous study by our research group (Liu et al., 2021). The retina is highly susceptible to ischemic stress for it is a metabolically demanding tissue in the body (Li et al., 2012). In the stroke model established by middle cerebral artery occlusion in rats, the blood flow of the ophthalmic artery was also impaired, resulting in retinal ischemia (Minhas et al., 2012). Moreover, the retina and the brain share similar physiological features, so the changes of retinal vessels are likely to be consistent with the pathological mechanisms of stroke, such as autoregulatory impairment, inflammation and endothelial dysfunction (Erskine and Herrera, 2014). Thus, the declines of cerebral blood flow in IS patients is associated with a decrease in retinal vascular perfusion. With regard to neural structures, the thinning of RNFLt in patients with IS was revealed in the present study, which was consistent with the results of another previous study (Park et al., 2013). Concordantly, we found a decrease in GCCt. Some experimental studies in rats have shown that the GCCt is reduced in stroke models. In the rat IS model, it can be observed the upregulation of stress-induced proteins such as hypoxia-inducible factor alpha and heat shock protein 70, c-Fos expression in the retina and a significant increase in the inflammatory molecules (IL-1, TNF etc.) (Stevens et al., 2002; Kalesnykas et al., 2008; Ritzel et al., 2016). These pathophysiological processes might be the mechanisms that underlie the decreased GCCt and RNFLt in patients with IS, since these inflammatory molecules have been shown to be cytotoxic to retinal neurons (Kumar, 2017).

Our study found that the parafoveal SCP VD was positively correlated with GCCt, and the whole image RPC VD was positively correlated with RNFLt. These findings suggest that the microvascular and neural damages of the retina may be synchronous in IS patients. In recent years, increased attention is being paid to the importance of neurovascular unit (NVU) dysfunction in the pathogenesis of IS (Chamorro et al., 2021). The NVU, a functional and structurally interdependent multicellular complex, comprises endothelial cells, pericytes, astrocytes, microglia, basement membrane, and neurons (Iadecola and Nedergaard, 2007; Zacchigna et al., 2008; del Zoppo, 2010). The structural and functional integrity of the cerebral NVU can be damaged after onset of IS. IS can cause a decrease in cerebral blood flow, leading to dysfunctional energy metabolism (Steliga et al., 2020). Damaged neurons release glutamate and triggering glutamatergic excitotoxicity further damage the neurons (Castillo et al., 1997; Choi, 2020). This is accompanied by the activation of oxidative stress along with an increase in production of reactive oxygen species which can cause damage to the NVU (Manzanero et al., 2013). At the same time, the release of proinflammatory cytokines and chemokines has a destructive effect on all components of the NVU (Fassbender et al., 1994). The blood-brain barrier, a structure composed of astrocytes, microglia, and pericytes, is also compromised (Zlokovic, 2008; Daneman et al., 2010; Kisler et al., 2020). In addition, the dysfunction of the neurovascular unit can lead to the activation of the immune system (Tang et al., 2007; Marsh et al., 2009; Abe et al., 2010). Toll-like receptor (TLR), the key receptor of innate immunity, can be expressed in neurons. The activation of TLR occurs following ischemic injury, and the TLR signaling plays a role in neurovascular injury. It is possible that the NVU is the key to link microvascular and neural damages in IS. The retina also displays similarities to the brain regarding its neural, inflammatory and immune responses to injury (Steele et al., 2008; London et al., 2013; Ritzel et al., 2016). Moreover, the retina also has its NVU, which may be one of the reasons of the significant correlations between retinal microvascular and neural structure alterations.

In our study, the SCP VD was correlated with the neural parameters, but no significant correlations between the DCP VD and the neural parameters were observed. The reason may be because the GCC and RNFL are located at a layer corresponding to the SCP (Braaf et al., 2012), and the GCCt and RNFLt can reflect the neural changes in the SCP. This finding also supports the theory of retinal NVU damages in IS.

Our study found that the SCP VD, DCP VD and RNFLt of PCS patients were lower than those of ACS patients. The occipital lobe is the visual cortical center which is supplied by the posterior circulation. Previous studies have shown that retinal blood flow and vessel density increases during light flicker and hyperoxia stimulation (Pechauer et al., 2015). We hypothesize that the visual cortex may transmit feedback to the retinal circulation during visual stimulation. Ischemic damage to the visual cortical center in PCS patients may lead to a reduction of the feedback to the retinal circulation, resulting in a decrease in retinal blood flow.

Diabetes is not only an important risk factor for IS, but also an important factor affecting the neurovascular structure of the retina. Most of the previous studies on the retinal changes of diabetic patients without diabetic retinopathy by OCTA showed a significant reduction in retinal vessel density (Cao et al., 2019; Zeng et al., 2019; Zhang et al., 2021). But Agra et al. found that there were no significant differences in retinal microvascular parameters between diabetic patients without diabetic retinopathy and controls (Agra et al., 2021). In our study, no significant differences were found in OCTA parameters between IS patients with or without diabetes, suggesting that diabetes might not affect the OCTA parameters in IS patients. Future studies are required to validate our findings.

The NVU plays a leading role in the pathophysiological process of IS, and the focus of stroke treatment has been shifted from a neuroprotective approach to neurovascular protection (Wang et al., 2021). The retina is an extension of the brain in terms of anatomical development. According to our results, the retinal NVU impairment might be also present in IS patients, and could be observed by OCTA. Future studies are required to investigate the association of cerebral NVU damage and retinal NVU alteration in IS. If the damage of NVU in the brain was synchronous to that in the retina, OCTA might be used to evaluate and monitor the cerebral neurovascular changes in IS patients.

We acknowledged several limitations to our research. Firstly, the cross-sectional design of this study restricts causal interpretations of the results. Our findings need to be validated by prospective studies. Secondly, this study included participants with diabetes or hypertension which may affect the microvascular and neural structure of retina, mainly because most IS patients have these comorbid diseases (Wang et al., 2013; Umemura et al., 2017). But there were no statistically significant differences in the history of diabetes and hypertension between the IS group and the control group, making it comparable. Finally, the extent of the lesions in IS patients should be included to investigate the relationship between the severity of stroke and retinal neurovascular changes in future studies.

## Conclusion

Our study demonstrated that microvascular hypoperfusion and neural structure thinning are characteristics of retinal neurovascular impairment in patients with IS. We also found significant correlations between retinal microvascular and neural alterations.

## Data Availability Statement

The raw data supporting the conclusions of this article will be made available by the corresponding authors, without undue reservation.

## Ethics Statement

The studies involving human participants were reviewed and approved by Institutional Review Board of the Guangdong Provincial People’s Hospital, Guangzhou, China [Registration Number: DREC2018148H(R1)]. Written informed consent to participate in this study was provided by the participants or their legal guardian/next of kin.

## Author Contributions

YH, XY, HY, YL, and BL: conception and design. YL, BL, XZ, YX, and YF: data collection and collation. YL, BL, GW, and ZD: data analysis and interpretation. YL, BL, and YH: drafting the article. YH, XY, and HY: data interpretation and final review of the manuscript. All authors revised and approved the submitted manuscript.

## Conflict of Interest

The authors declare that the research was conducted in the absence of any commercial or financial relationships that could be construed as a potential conflict of interest.

## Publisher’s Note

All claims expressed in this article are solely those of the authors and do not necessarily represent those of their affiliated organizations, or those of the publisher, the editors and the reviewers. Any product that may be evaluated in this article, or claim that may be made by its manufacturer, is not guaranteed or endorsed by the publisher.
