# Supplementary material for: Retinal Neurovascular Changes in Patients With Ischemic Stroke Investigated by Optical Coherence Tomography Angiography
Source: Front Aging Neurosci. 2022 Jul 4;14:834560. doi: 10.3389/fnagi.2022.834560 (PMC9289443; doi:10.3389/fnagi.2022.834560)
Supplement: Supplementary file 1 [file Table_1.DOCX]

**Supplementary Online Content**

**Table S1**. Comparison of OCTA parameters in the Ischemic Stroke Patients

|  | duration | | *p* value |
| --- | --- | --- | --- |
|  | ＜median | ≥median |  |
| **Retinal microvascular parameters** |  |  |  |
| SCP VD (%) |  |  |  |
| Whole Image | 47.74(4.31) | 47.94(3.71) | 0.754 |
| Parafovea | 49.42(5.25) | 50.26(4.66) | 0.287 |
| Perifovea | 48.59(4.39) | 48.66(3.93) | 0.919 |
| DCP VD (%) |  |  |  |
| Whole Image | 47.98(6.05) | 48.62(5.03) | 0.468 |
| Parafovea | 52.34(4.81) | 53.30(3.91) | 0.166 |
| Perifovea | 49.02(6.68) | 49.67(5.74) | 0.507 |
| RPC VD (%) |  |  |  |
| Whole Image | 49.13(2.30) | 49.00(2.65) | 0.738 |
| Peripapillary | 51.25(2.55) | 51.23(2.61) | 0.956 |
| S-Hemi | 51.64(2.74) | 51.41(2.97) | 0.609 |
| I-Hemi | 51.11(3.33) | 51.06(2.80) | 0.930 |
| **Retinal neural parameters** |  |  |  |
| GCC |  |  |  |
| Whole Image, μm | 98.05(8.23) | 99.39(8.71) | 0.320 |
| Parafovea, μm | 104.89(9.46) | 105.71(10.05) | 0.599 |
| perifovea, μm | 97.81(8.40) | 99.75(8.63) | 0.155 |
| FLV, % | 1.06(1.10) | 1.38(1.46) | 0.115 |
| GLV, % | 3.23(3.06) | 2.75(2.76) | 0.300 |
| RNFL(μm) |  |  |  |
| Mean | 112.32(13.83) | 114.23(11.19) | 0.340 |
| S-Hemi | 112.99(13.57) | 115.16(10.28) | 0.259 |
| I-Hemi | 111.12(14.78) | 112.98(13.00) | 0.402 |

Abbreviations: VD, vessel density; SCP, superficial capillary plexus; DCP, deep capillary plexus; RPC, radial peripapillary capillaries; GCC, ganglion cell complex; RNFL, retinal nerve fibre layer; FLV, focal loss volume; GLV, global loss volume; S-Hemi, superior-hemifield; I-Hemi, inferior-hemifield.

The ischemic stroke patients were divided into two groups based on the median of the duration. The OCTA parameters were not significantly different between the ischemic stroke patients with shorter time interval and those with longer time interval.

* p < 0.05 was statistically significant by independent, two-tailed student’s t tests.

**Table S2**. Correlations between OCTA parameters and Duration of Ischemic Stroke

|  | *r* | *p* value |
| --- | --- | --- |
| **Retinal microvascular parameters** |  |  |
| SCP VD (%) |  |  |
| Whole Image | 0.011 | 0.886 |
| Parafovea | 0.083 | 0.299 |
| Perifovea | 0.009 | 0.914 |
| DCP VD (%) |  |  |
| Whole Image | 0.096 | 0.229 |
| Parafovea | 0.129 | 0.105 |
| Perifovea | 0.101 | 0.204 |
| RPC VD (%) |  |  |
| Whole Image | -0.033 | 0.676 |
| Peripapillary | -0.014 | 0.859 |
| S-Hemi | -0.024 | 0.765 |
| I-Hemi | -0.046 | 0.563 |
| **Retinal neural parameters** |  |  |
| GCC |  |  |
| Whole Image, μm | 0.075 | 0.350 |
| Parafovea, μm | 0.063 | 0.429 |
| perifovea, μm | 0.095 | 0.235 |
| FLV, % | 0.030 | 0.705 |
| GLV, % | -0.087 | 0.275 |
| RNFL(μm) |  |  |
| Mean | 0.024 | 0.762 |
| S-Hemi | 0.044 | 0.580 |
| I-Hemi | 0.006 | 0.938 |

Abbreviations: VD, vessel density; SCP, superficial capillary plexus; DCP, deep capillary plexus; RPC, radial peripapillary capillaries; GCC, ganglion cell complex; RNFL, retinal nerve fibre layer; FLV, focal loss volume; GLV, global loss volume; S-Hemi, superior-hemifield; I-Hemi, inferior-hemifield.

OCTA parameters in the ischemic stroke group were not correlated to the time interval between ischemic stroke attacks and OCTA examination.

* p < 0.05 was statistically significant by Spearman’s correlation analysis.

**TableS3.** The Correlation between Microvascular Parameters and Neural Parameters in Control Group

|  | Correlated Parameters | *r* ^#^ | *p* value^##^ |
| --- | --- | --- | --- |
| SCP-Parafovea | Whole Image GCC | 0.228 | 0.022^*^ |
|  | Parafovea GCC | 0.256 | 0.010^*^ |
|  | Perifovea GCC | 0.223 | 0.025^*^ |
|  | GCC-FLV | 0.023 | 0.821 |
|  | GCC-GLV | 0.020 | 0.846 |
| DCP-Parafovea | Whole Image GCC | 0.029 | 0.777 |
|  | Parafovea GCC | 0.092 | 0.360 |
|  | Perifovea GCC | 0.038 | 0.706 |
|  | GCC-FLV | 0.062 | 0.537 |
|  | GCC-GLV | 0.157 | 0.116 |
| RPC-Whole Image | Mean RNFL | 0.101 | 0.316 |
|  | S-Hemi RNFL | 0.057 | 0.572 |
|  | I-Hemi RNFL | 0.087 | 0.385 |

Abbreviations: SCP, superficial capillary plexus; DCP, deep capillary plexus; RPC, radial peripapillary capillaries; GCC, ganglion cell complex; RNFL, retinal nerve fibre layer; FLV, focal loss volume; GLV, global loss volume; S-Hemi, superior-hemifield; I-Hemi, inferior-hemifield.

^#^ *r* indicates the partial correlation coefficient between microvascular parameters and neural parameters.

^##^Adjusted for sex, SBP, DBP, history of smoking, HbA1c, CHOL, HDL, BCVA of enrolled eyes.

**p*<0.05 is considered statistically significant.

**Table S4** . Risk factors for IS using binary logistic regression analysis

|  | OR (95% CI) | *p* value |
| --- | --- | --- |
| **Retinal microvascular parameters** |  |  |
| SCP VD (%) |  |  |
| Whole Image | 0.873(0.813,0.937) | ＜0.001^*^ |
| Parafovea | 0.869(0.817,0.926) | ＜0.001^*^ |
| Perifovea | 0.875(0.817,0.937) | ＜0.001^*^ |
| DCP VD (%) |  |  |
| Whole Image | 0.903(0.862,0.947) | ＜0.001^*^ |
| Parafovea | 0.848(0.793,0.906) | ＜0.001^*^ |
| Perifovea | 0.911(0.873,0.951) | ＜0.001^*^ |
| RPC VD (%) |  |  |
| Whole Image | 0.884(0.797,0.981) | 0.020^*^ |
| Peripapillary | 0.883(0.803,0.972) | 0.011^*^ |
| S-Hemi | 0.941(0.864,1.025) | 0.163 |
| I-Hemi | 0.881(0.808,0.962) | 0.005^*^ |
| **Retinal neural parameters** |  |  |
| GCC |  |  |
| Whole Image, μm | 0.956(0.926,0.988) | 0.007^*^ |
| Parafovea, μm | 0.969(0.941,0.997) | 0.031^*^ |
| perifovea, μm | 0.957(0.928,0.987) | 0.006^*^ |
| FLV, % | 1.795(1.317,2.446) | ＜0.001^*^ |
| GLV, % | 1.249(1.113,1.402) | ＜0.001^*^ |
| RNFL(μm) |  |  |
| Mean | 0.982(0.962,1.002) | 0.073 |
| S-Hemi | 0.985(0.966,1.005) | 0.145 |
| I-Hemi | 0.978(0.959,0.996) | 0.020^*^ |

Abbreviations: VD, vessel density; SCP, superficial capillary plexus; DCP, deep capillary plexus; RPC, radial peripapillary capillaries; GCC, ganglion cell complex; RNFL, retinal nerve fibre layer; FLV, focal loss volume; GLV, global loss volume; S-Hemi, superior-hemifield; I-Hemi, inferior-hemifield.

* p < 0.05 was statistically significant.

**Table S5**.Comparison of OCTA Parameters between ACS and PCS

|  | ACS(n=104) | PCS(n=55) | *p* value^#^ |
| --- | --- | --- | --- |
| **Retinal microvascular parameters** |  |  |  |
| SCP VD (%), mean(SD) |  |  |  |
| Whole Image | 48.4(3.7) | 46.7(4.3) | 0.018^*^ |
| Parafovea | 50.3(4.8) | 49.0(5.1) | 0.145 |
| Perifovea | 49.3(3.8) | 47.4(4.5) | 0.009^*^ |
| DCP VD (%), mean(SD) |  |  |  |
| Whole Image | 49.0(5.5) | 47.0(5.5) | 0.042^*^ |
| Parafovea | 53.4(4.3) | 51.8(4.3) | 0.050 |
| Perifovea | 49.5(6.0) | 48.8(6.8) | 0.009^*^ |
| RPC VD (%), mean(SD) |  |  |  |
| Whole Image | 49.2(2.4) | 48.8(2.5) | 0.619 |
| Peripapillary | 51.3(2.5) | 51.0(2.7) | 0.797 |
| S-Hemi | 51.7(2.8) | 51.2(2.9) | 0.530 |
| I-Hemi | 51.3(3.0) | 50.8(3.1) | 0.570 |
| **Retinal neural parameters** |  |  |  |
| GCC, mean(SD) |  |  |  |
| Whole Image, μm | 99.7(8.4) | 96.9(8.5) | 0.043^*^ |
| Parafovea, μm | 105.8(9.8) | 104.3(9.6) | 0.282 |
| perifovea, μm | 100.0(8.2) | 96.5(8.7) | 0.014^*^ |
| FLV, % | 1.1(1.1) | 1.5(1.6) | 0.140 |
| GLV, % | 2.7(2.9) | 3.5(2.9) | 0.155 |
| RNFL(μm), mean(SD) |  |  |  |
| Mean | 114.0(13.1) | 112.0(11.4) | 0.530 |
| S-Hemi | 114.6(12.5) | 113.2(11.1) | 0.656 |
| I-Hemi | 113.1(14.3) | 110.2(13.0) | 0.317 |

Abbreviations: ACS, anterior circulation stroke; PCS, posterior circulation stroke; VD, vessel density; SCP, superficial capillary plexus; DCP, deep capillary plexus; RPC, radial peripapillary capillaries; GCC, ganglion cell complex; RNFL, retinal nerve fibre layer; FLV, focal loss volume; GLV, global loss volume; S-Hemi, superior-hemifield; I-Hemi, inferior-hemifield.

^#^Adjusted for sex, SBP, DBP, history of smoking, HbA1c, CHOL, HDL, BCVA of enrolled eyes.

* *p*<0.05 is considered statistically significant.

**Table S6.** Comparison of OCTA Parameters in the IS Group Stratified by Gender

|  | Males | Females | *p* value^#^ |
| --- | --- | --- | --- |
| **Retinal microvascular parameters** |  |  |  |
| SCP VD (%), mean(SD) |  |  |  |
| Whole Image | 47.7(4.0) | 48.2(4.2) | 0.371 |
| Parafovea | 49.6(5.1) | 50.7(4.6) | 0.259 |
| Perifovea | 48.5(4.1) | 48.9(4.4) | 0.358 |
| DCP VD (%), mean(SD) |  |  |  |
| Whole Image | 48.5(5.4) | 47.8(6.0) | 0.744 |
| Parafovea | 52.7(4.3) | 53.4(4.5) | 0.135 |
| Perifovea | 49.5(6.0) | 48.8(6.8) | 0.694 |
| RPC VD (%), mean(SD) |  |  |  |
| Whole Image | 48.9(2.6) | 49.5(2.2) | 0.200 |
| Peripapillary | 51.1(2.7) | 51.6(2.2) | 0.277 |
| S-Hemi | 51.4(2.9) | 52.0(2.5) | 0.107 |
| I-Hemi | 51.0(3.2) | 51.4(2.7) | 0.392 |
| **Retinal neural parameters** |  |  |  |
| GCC, mean(SD) |  |  |  |
| Whole Image, μm | 98.7(8.7) | 98.8(8.0) | 0.574 |
| Parafovea, μm | 105.5(9.8) | 104.7(9.7) | 0.164 |
| perifovea, μm | 98.8(8.6) | 98.7(8.5) | 0.699 |
| FLV, % | 1.2(1.3) | 1.3(1.3) | 0.970 |
| GLV, % | 2.9(2.9) | 3.3(2.9) | 0.854 |
| RNFL(μm), mean(SD) |  |  |  |
| Mean | 112.6(13.0) | 115.5(10.9) | 0.228 |
| S-Hemi | 113.5(12.6) | 116.0(10.0) | 0.201 |
| I-Hemi | 111.4(14.2) | 114.2(12.8) | 0.421 |

Abbreviations: VD, vessel density; SCP, superficial capillary plexus; DCP, deep capillary plexus; RPC, radial peripapillary capillaries; GCC, ganglion cell complex; RNFL, retinal nerve fibre layer; FLV, focal loss volume; GLV, global loss volume; S-Hemi, superior-hemifield; I-Hemi, inferior-hemifield.

^#^Adjusted for sex, SBP, DBP, history of smoking, HbA1c, CHOL, HDL, BCVA of enrolled eyes.

* *p*<0.05 is considered statistically significant.

**Table S7.** Comparison of OCTA Parameters in the Control Group Stratified by Gender

|  | Males | Females | *p* value^#^ |
| --- | --- | --- | --- |
| **Retinal microvascular parameters** |  |  |  |
| SCP VD (%), mean(SD) |  |  |  |
| Whole Image | 49.6(3.6) | 49.9(3.6) | 0.208 |
| Parafovea | 52.4(4.2) | 52.7(4.0) | 0.246 |
| Perifovea | 50.3(3.7) | 50.8(3.6) | 0.140 |
| DCP VD (%), mean(SD) |  |  |  |
| Whole Image | 51.0(5.3) | 51.7(5.8) | 0.250 |
| Parafovea | 54.6(3.9) | 56.3(3.7) | 0.063 |
| Perifovea | 52.4(5.8) | 53.1(6.3) | 0.219 |
| RPC VD (%), mean(SD) |  |  |  |
| Whole Image | 49.5(2.4) | 49.9(2.3) | 0.333 |
| Peripapillary | 51.8(2.7) | 52.3(2.9) | 0.635 |
| S-Hemi | 51.6(2.9) | 52.3(3.0) | 0.321 |
| I-Hemi | 51.9(2.7) | 52.3(2.8) | 0.565 |
| **Retinal neural parameters** |  |  |  |
| GCC, mean(SD) |  |  |  |
| Whole Image, μm | 100.6(7.0) | 101.9(6.5) | 0.286 |
| Parafovea, μm | 108.4(7.3) | 107.2(7.0) | 0.528 |
| perifovea, μm | 100.2(7.4) | 102.6(6.9) | 0.085 |
| FLV, % | 0.6(0.5) | 0.8(0.7) | 0.227 |
| GLV, % | 1.6(1.9) | 1.8(1.8) | 0.883 |
| RNFL(μm), mean(SD) |  |  |  |
| Mean | 115.0(12.0) | 116.8(12.3) | 0.431 |
| S-Hemi | 115.3(13.3) | 117.1(12.8) | 0.420 |
| I-Hemi | 114.2(12.7) | 117.1(11.7) | 0.404 |

Abbreviations: VD, vessel density; SCP, superficial capillary plexus; DCP, deep capillary plexus; RPC, radial peripapillary capillaries; GCC, ganglion cell complex; RNFL, retinal nerve fibre layer; FLV, focal loss volume; GLV, global loss volume; S-Hemi, superior-hemifield; I-Hemi, inferior-hemifield.

^#^Adjusted for sex, SBP, DBP, history of smoking, HbA1c, CHOL, HDL, BCVA of enrolled eyes.

* *p*<0.05 is considered statistically significant.
